# Potential CD8+ T Cell Cross-Reactivity Against SARS-CoV-2 Conferred by Other Coronavirus Strains

**DOI:** 10.3389/fimmu.2020.579480

**Published:** 2020-11-05

**Authors:** Chloe H. Lee, Mariana Pereira Pinho, Paul R. Buckley, Isaac B. Woodhouse, Graham Ogg, Alison Simmons, Giorgio Napolitani, Hashem Koohy

**Affiliations:** ^1^MRC Human Immunology Unit, Medical Research Council (MRC) Weatherall Institute of Molecular Medicine (WIMM), John Radcliffe Hospital, University of Oxford, Oxford, United Kingdom; ^2^Radcliffe Department of Medicine, University of Oxford, Oxford, United Kingdom; ^3^MRC WIMM Centre For Computational Biology, MRC Weatherall Institute of Molecular Medicine, John Radcliffe Hospital, University of Oxford, Oxford, United Kingdom; ^4^Translational Gastroenterology Unit, John Radcliffe Hospital, Oxford, United Kingdom; ^5^NIHR Oxford Biomedical Research Centre, Oxford, United Kingdom

**Keywords:** cross-reactivity, antigen presentation, predict immunogenicity, epitopes, CD8+ T cell recognition, COVID-19, SARS-CoV-2

## Abstract

While individuals infected with coronavirus disease 2019 (COVID-19) manifested a broad range in susceptibility and severity to the disease, the pre-existing immune memory to related pathogens cross-reactive against SARS-CoV-2 can influence the disease outcome in COVID-19. Here, we investigated the potential extent of T cell cross-reactivity against severe acute respiratory syndrome coronavirus 2 (SARS-CoV-2) that can be conferred by other coronaviruses and influenza virus, and generated an *in silico* map of public and private CD8+ T cell epitopes between coronaviruses. We observed 794 predicted SARS-CoV-2 epitopes of which 52% were private and 48% were public. Ninety-nine percent of the public epitopes were shared with SARS-CoV and 5.4% were shared with either one of four common coronaviruses, 229E, HKU1, NL63, and OC43. Moreover, to assess the potential risk of self-reactivity and/or diminished T cell response for peptides identical or highly similar to the host, we identified predicted epitopes with high sequence similarity with human proteome. Lastly, we compared predicted epitopes from coronaviruses with epitopes from influenza virus deposited in IEDB, and found only a small number of peptides with limited potential for cross-reactivity between the two virus families. We believe our comprehensive *in silico* profile of private and public epitopes across coronaviruses would facilitate design of vaccines, and provide insights into the presence of pre-existing coronavirus-specific memory CD8+ T cells that may influence immune responses against SARS-CoV-2.

## Introduction

Faced by unprecedent health and economic crisis from the coronavirus disease 2019 (COVID-19), the scientific community is pushing forward with efforts to develop vaccines and treatments to mitigate its impact. While the severity of symptoms have been reported to be associated with age, gender, and comorbidities such as cardiovascular diseases and chronic respiratory diseases (1), the underlying mechanism of broad variation in susceptibility and severity to COVID-19 is not fully understood (2). It is however accepted that an altered immune response is a key contributor to pathology (3, 4), and the balance between generation of protective and pathological immune responses by the host may be a vital factor governing the disease outcome.

As immune memory by related pathogens has shown to help reduce severity and spread of the diseases (5–7), pre-existing immunity through cross-reactivity to familial coronavirus strains may provide individuals with protection or enhanced susceptibility against the severe acute respiratory syndrome coronavirus 2 (SARS-CoV-2) without prior exposure (8–10). Therefore, we aim to characterize the potential for the existing immune memory by other coronaviruses and influenza virus to fight against SARS-CoV-2 and further identify targets for developing a “universal” vaccine against coronaviruses.

The strains infecting humans belong to either alpha and beta genera of coronavirus. The alphacoronavirus contains human coronavirus 229E (HCoV-229E) and HCoV-NL63, while the betacoronavirus contains HCoV-OC43, and HCoV-HKU1, Middle East respiratory syndrome coronavirus (MERS-CoV), SARS-CoV, and SARS-CoV-2 (11). It is known that NL63, 229E, OC43, and HKU1 usually cause only mild to moderate symptoms such as cough, runny nose, fever, and sore throat like the common cold (12), whereas MERS-CoV and SARS-CoV cause more severe symptoms including respiratory tract disease.

In this study, we investigated the level of T cell antigen cross-reactivity across the seven alpha and betacoronavirus strains, evaluated the risk of self-reactivity from SARS-CoV-2 predicted epitopes, and identified targets for vaccine developments against coronavirus and influenza virus. We first predicted the potential of peptides to be presented by 10 prevalent HLA alleles and eliciting CD8+ T cell responses, and generated a comprehensive *in silico* profile of public and private predicted epitopes. We also expanded the map of cross-reactivity from exact matching peptides to those with a high sequence similarity, resulting in addition of 264 and 283 public SARS-CoV-2 predicted epitopes by allowing one and two amino acid mismatches, respectively. Moreover, to assess the risk of self-reactivity and immunopathology, we compared SARS-CoV-2 predicted epitopes with human proteome sequences and detected 10 predicted epitopes that are single amino acid variant from their counterparts in the human proteome. Lastly, to explore the potential for development of vaccines against coronavirus and influenza virus, we compared our list of predicted epitopes from coronaviruses with epitopes from influenza virus deposited in Immune Epitope Database and Analysis Resource (IEDB) and detected only a limited number of epitopes with a modest sequence similarity that are shared across multiple coronavirus strains and influenza.

## Methods

### Retrieval of Coronavirus Proteome Sequences

The proteome sequences of coronavirus strains were obtained from NCBI. The reference numbers for these sequences are NC_002645.1 (229E), NC_006577.2 (HKU1), NC_019843.3 (MERS-CoV), NC_005831.2 (NL63), NC_006213.1 (OC43), NC_004718.3 (SARS-CoV) and NC_045512.2 (SARS-CoV-2).

### Sequence Alignment and Phylogenetic Tree of Encoded Proteins in Coronaviruses

For multiple sequence alignment and phylogenetic tree generation, the open reading frames were grouped by their encoded proteins—spike protein (S), envelope protein (E), membrane protein (M), nucleocapsid protein (N), replicase polyprotein (Orf1ab), and other encoded regions (Other)—prior to analysis. The multiple sequence alignment was conducted using “msa” function from R msa v1.4.3 package and visualized by “msaPrettyPrint” function. The phylogenetic tree was produced by plotting “identity” distance generated by “dist.alignment” function from R seqinr v3.6.1 package.

### Peptide Generation From Proteome Sequences

Each encoded proteins were fragmented into 9-mer peptides by scanning the proteome with a window of nine amino acids and step length of one amino acid. For strains containing two open reading frames annotated with the same functional protein e.g. 229E, MERS-CoV, SARS-CoV, and SARS-CoV-2 having two open reading frames annotated for Orf1ab, 9-mer peptides were generated from both encoded proteins and unique set of peptides were selected for subsequent analysis.

### MHC Presentation Prediction

The antigen presentation of MHC was predicted using NetMHCpan v4.0 (13), a model trained on binding affinity and eluted ligand data, against HLA-A*0101, 0201, 0301, 2402, HLA-B*0702, 4001, 0801, and HLA-C*0702, 0401, 0701 alleles. Peptides with rank score <=2.0 were categorized as positive HLA-binder.

### Immunogenicity Prediction

The immunogenicity potential was predicted by R package Repitope for peptides that passed NetMHCpan filtering i.e. those predicted to bind at least one HLA allele. The Repitope utilizes amino acid descriptors and TCR-peptide contact potential profiling (CPP)-based features to label immunogenicity. The Repitope package was retrieved from GitHub repository (https://github.com/masato-ogishi/Repitope.git).

After feature computation and feature selection, we utilized the published Repitope “MHCI_Human” model to extrapolate probabilistic immunogenicity scores for our dataset and made binary classification of immunogenicity. This classification was based on a threshold computed from the ROC curves of the MHCI_Human immunogenicity prediction model and was calculated using the youden index that maximizes *1-sensitivity+specificity*. Probabilistic scores were extracted from the original model for a subset of peptides that were identical to peptides in the model’s training dataset.

### Visualization of Private and Public Peptides

The conservation of peptides across coronavirus strains before and after MHC binding and immunogenicity prediction were visualized by “venn” function from R venn v1.9 package and upset function from R UpSetR v1.4.0 package. To identify shared peptides with up to two amino acid tolerance, the best matching peptides were identified by “pairwiseAlignment” from Biostrings v2.40.2 package using BLOSUM62 matrix, gapOpening of 100, and gapExtension of 100, followed by hamming distance to filter only peptide pairs with less than or equal to two amino acid difference.

### Sequence Similarity With Human Proteome and Epitopes Deposited in IEDB

The sequence similarity of peptides derived from SARS-CoV-2 with human proteome counterparts was computed by first identifying best global-local alignment by “pairwiseAlignment” function from Biostrings v2.40.2 package with a high gap penalty, gapOpening of 100 and gapExtension of 100. The number of mismatches between best aligned pair was computed by hamming distance using “stringdist” function from R stringdist v0.9.5.5 package. Similarly, the sequence similarity between coronavirus peptides and epitopes from IEDB was compared by global-local alignment following by computing hamming distance to find the best matching pairs. In evaluating the accuracy of predicted epitopes with epitopes deposited in IEDB, the epitopes with matching sequence of predicted epitopes were retrieved.

## Results

### Shared Predicted Epitopes Among Coronavirus Strains

**Figure 1:**
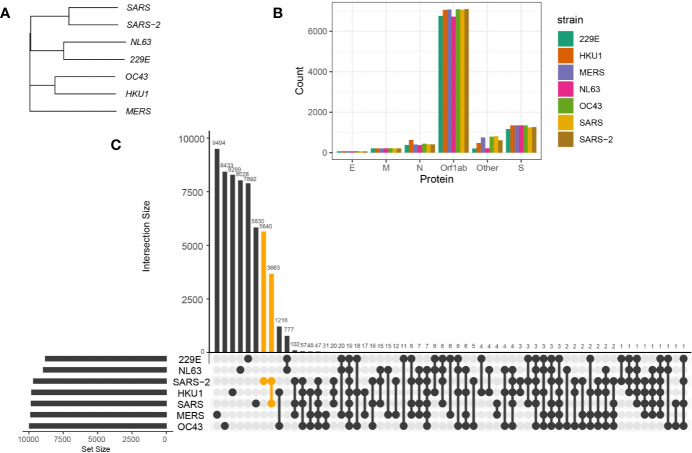
Sequence homology between coronavirus strains. **(A)** Phylogenetic tree of spike protein sequences between NL63, 229E, OC43, HKU1, MERS-CoV, SARS-CoV, and SARS-CoV-2 strains. **(B)** Number of 9-mer peptides generated from each coronavirus strains grouped by functional proteins, envelope protein (E), membrane protein (M), nucleocapsid protein (N) and replicase polyprotein (Orf1ab), spike protein (S), and other encoded proteins (Other). **(C)** Number of shared and private 9-mer peptides between coronavirus strains. The UpSet plot illustrates interaction of 9-mer peptides between seven coronavirus strains. The number of 9-mer peptides unique from SARS-CoV-2 or shared with SARS-CoV are colored in orange.

To first evaluate the homology of proteome sequences among alpha- and betacoronavirus strains, we conducted sequence alignment and generated a phylogenetic tree of encoded proteins between NL63, 229E, OC43, HKU1, MERS-CoV, SARS-CoV, and SARS-CoV-2 (see *Methods*). Based on sequence alignments (example alignment of spike protein illustrated in **Supplementary Figure 1**) and phylogenetic trees of encoded proteins, the alpha- and betacoronavirus strains shared a high sequence similarity within their own genera, i.e. higher similarity between NL63 and 229E than with betacoronaviruses. In particular, for betacoronavirus strains, phylogenetic analysis showed higher similarity between OC43 and HKU1, and between SARS-CoV and SARS-CoV-2. Notably, MERS-CoV showed relatively distinct proteome sequences to all other coronavirus strains included in this study (**Figure 1A** for spike protein and **Supplementary Figure 2** for other encoded proteins).

To identify the conserved 9-mer peptides across coronavirus strains, we first generated 9-mer peptides from encoded proteins of coronavirus strains (**Figure 1B**) and detected public peptides with identical matches (**Figure 1C**). Notably, there were 3,663 shared 9-mer peptides between SARS-CoV and SARS-CoV-2. Given the longest open reading frame of replicase polyproteins (Orf1ab) with an average of 7,002 amino acids across coronavirus strains, many public peptides were derived from Orf1ab, with 19 peptides shared across all studied strains (**Figure 3A**, first panel).

**Figure 2:**
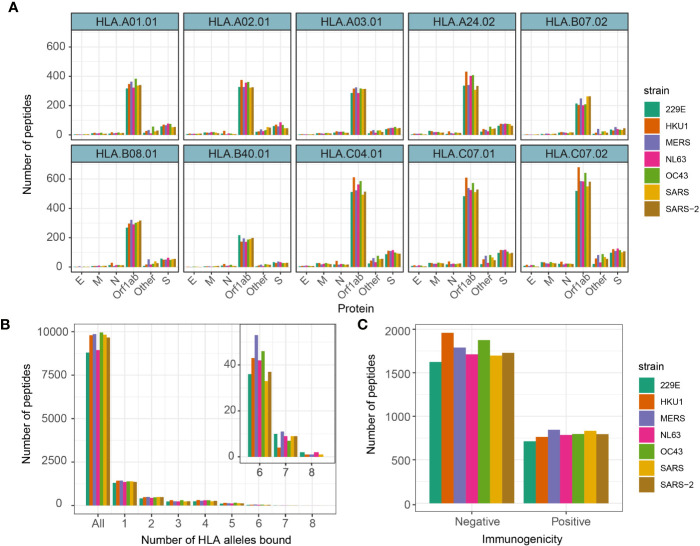
Number of 9-mer peptides predicted to bind HLA alleles and immunogenic. **(A)** Number of 9-mer peptides from each coronavirus strains predicted to bind annotated HLA alleles. **(B)** Number of peptides predicted to bind equal to specified number of HLA alleles. **(C)** Number of peptides predicted to productively interact with TCRs by Repitope prediction.

We then investigated antigen presentation potential of 9-mer peptides for 10 most prevalent HLA alleles corresponding to MHC class I (HLA-A, HLA-B, and HLA-C alleles). These include HLA-A*0101, 0201, 0301, 2402, HLA-B*0702, 4001, 0801, and HLA-C*0702, 0401, 0701 (14). Prevalence of these HLA alleles in the UK and US population has been referenced in **Supplementary Figure 3A**. The MHC presentation was predicted by NetMHCpan v4.0 (13) (see *Methods*). Generally, there was a relatively high number of peptides predicted to bind HLA-C alleles while HLA-B alleles had the lowest number of predicted binders (**Figure 2A**). We identified on average 2,559 (SD = 120) peptides predicted to bind at least one HLA allele (**Figure 2B**), which is ~21% of total number of 9-mer peptides across different strains. Of interest, there were 66 peptides from seven strains predicted to bind at least seven different HLA alleles, of which nine peptides were derived from SARS-CoV-2. The peptides predicted to bind 8 HLA alleles and their derived proteins are listed in **Supplementary Figure 3B**.

Although viral antigen presentation is a vital step in triggering immune responses, not all MHC binding peptides are immunogenic. We therefore set out to predict T cell immunogenicity, i.e. the ability of a peptide presented by an MHC molecule to productively interact with a T cell receptor, of all peptides predicted to bind at least one HLA allele. In an ongoing unpublished study, we have benchmarked the existing immunogenicity predicting models and as a result recognized a recently published model called *Repitope* (15) as the best performing existing model to predict immunogenicity of viral epitopes.

We therefore utilized Repitope (see *Methods*) and identified in total 4,894 out of 16,096 (~30%) unique predicted binders to be immunogenic (subsequently referred to as predicted epitopes), and the proportion of such predicted epitopes was comparable across different strains (**Figure 2C**, **Supplementary Figure 4**). On average, we detected 429 (SD = 26) epitopes predicted to bind at least one HLA type and be immunogenic. The full list of predicted immunogenic and nonimmunogenic HLA-binders are provided in **Supplementary Table 1**.

**Figure 3:**
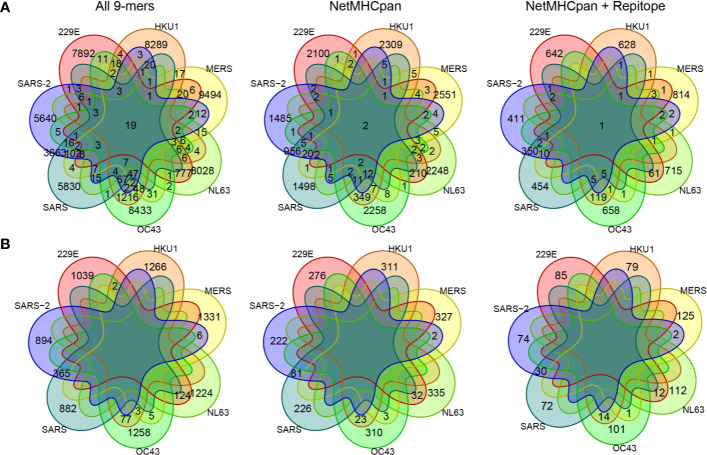
Comparing private and public 9-mer peptides from a complete set of peptides to after MHC presentation prediction by NetMHCpan and immunogenicity prediction by Repitope. Venn diagram is colored by strains. **(A)** Peptides derived from all encoded proteins. **(B)** Peptides derived from spike protein only.

With the pool of predicted epitopes, we generated Venn diagrams to illustrate private and public epitopes across different coronavirus strains (**Figure 3**, **Supplementary Figure 5**). From a total of 794 predicted epitopes from SARS-CoV-2, 411 (/794, ~52%) were private while the remaining 383 (/794, ~48%) were public of which 379 (/383, ~99%) were shared with SARS-CoV and 21 (/383, ~5.4%) were shared with either one of four common coronaviruses, 229E, HKU1, NL63, and OC43. We detected only one predicted epitope—SLAIDAYPL derived from Orf1ab—public across all strains. Given the long sequence of Orf1ab protein, many of the public epitopes were derived from Orf1ab. This analysis suggests a large extent of CD8+ T cell cross-reactivity between SARS-CoV-2 and SARS-CoV but limited number of targets with other common coronaviruses.

Here, we conducted predictions of HLA binding and immunogenicity on 9-mer peptides as they were shown most prevalent for MHC binding compared to peptides of other lengths (16). However, it is now clear that peptides between lengths of 8–15aa can bind to HLA-I molecules and elicit immune responses (17, 18). Therefore, in parallel to the analysis of 9-mer peptides, we studied publicness of 10-mer predicted epitopes (**Supplementary Figure 6A**, **Supplementary Table 2**). In general given the longer sequences, there were less public peptides across coronavirus strains. Notably, as peptide length affects binding of HLA alleles differently (17), fewer peptides were predicted to bind HLA-B*08:01, HLA-C*04:01, HLA-C*07:01, HLA-C*07:02 (**Supplementary Figure 6B**). Peptides of other lengths can be readily investigated in the same manner.

### Validating Predictions by Epitopes Deposited in IEDB

For a validation of predicted epitopes, we have compared the list of predicted epitopes with epitopes characterized by *in vitro* T cell assays and deposited in Immune Epitope Database and Analysis Resource (IEDB). We retrieved all 7,869 linear epitopes in IEDB (as of 05-05-2020), presented on MHC-I, reported positive in T cell assays and have human as the host organism then compared with the list of 4,894 unique predicted epitopes from SARS-CoV-2.

From the 7,869 IEBD immunogenic peptides, there were 34 unique peptides derived from coronavirusstrains, one from OC43, two from 229E, and the remaining from SARS-CoV. We identified 25 unique9-mer predicted epitopes from four coronavirus strains, 229E, NL63, SARS-CoV, and SARS-CoV-2 having matching pattern with 24 IEDB peptides (**Table 1**). The matching epitopes from NL63 and SARS-CoV-2 found in our analysis were shared with 229E and SARS-CoV respectively (**Supplementary Table 3**). Consequently, all 229E and NL63 predicted epitopes were matching with 229E IEBD peptides, whereas all SARS-CoV and SARS-CoV-2 predicted epitopes were matching with SARS-CoV IEBD peptides. None of our predicted epitopes matched with peptides derived from non-coronavirus microorganisms, suggesting a restricted potential for cross-reactivity between coronavirus and other virus strains.

**Table 1:** Number of unique peptides by strain having matching pattern with immunogenic peptides deposited in IEDB.

Strain	Number of predicted 9-mer epitopes with matches in IEDB	Number of IEDB peptide matches(total for each strain)
229E	2	2 (2)
NL63	1*	0 (0)
OC43	0	0 (1)
SARS	23	22 (31)
SARS-2	16^	0 (0)

Multiple 9-mer peptides may have matching pattern with a single IEDB epitope and vice versa. *Match with 229E IEDB peptide; ^Match with SARS peptides.

Notably, 22 out of 31 (71%) SARS-derived immunogenic peptides in IEDB matched with our 23 SARS-derived 9-mer predicted epitopes (**Table 1**). Among the nine false negative SARS-derived IEDB peptides, seven were predicted MHC nonbinders and two were predicted non-immunogenic by our analysis. Despite the false negatives, 71% of true positives were retrieved from the shortlisted predicted epitopes, indicating a higher probability of identifying immunogenic peptides to facilitate targeted identification and validation of vaccine candidates.

### Public Epitopes Between SARS-CoV-2 and Other Coronavirus Strains by High Sequence Similarity

**Figure 4:**
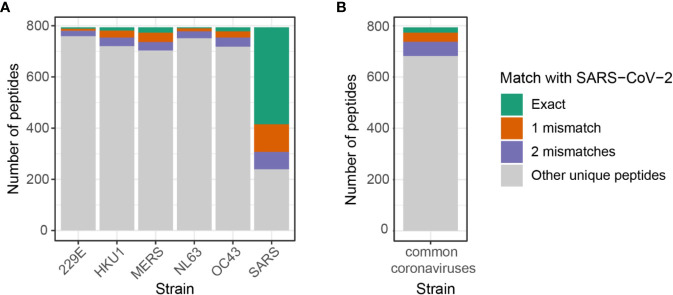
Number of shared predicted epitopes between SARS-CoV-2 and other coronavirus strains by allowing up to two mismatches. **(A)** Map of public peptides out of 794 SARS-CoV-2 predicted epitopes expanded by allowing up to two amino acids difference. Note that it includes duplicated peptides that may be shared across coronavirus strains, i.e. peptides shared across SARS-CoV-2, SARS-CoV, and MERS-CoV are counted in both SARS and MERS. Numbers can be retrieved from **Supplementary Figure 7**. **(B)** Unique predicted epitopes from four common coronaviruses, 229E, HKU1, NL63, and OC43, shared with 794 SARS-CoV-2 predicted epitopes. Numbers can be retrieved from **Supplementary Figure 8**.

In addition to public peptides by exact matches, predicted epitopes with a high sequence similarity may also trigger cross-reactivity across coronavirus strains. Here, we expanded the previous set of public predicted epitopes of SARS-CoV-2 to include those with up to two amino acid mismatches (**Figure 4A**, **Supplementary Table 4**). In addition to 383 predicted epitopes from SARS-CoV-2 shared with other coronavirus strains, there can be an increase of 173 and 159 unique public epitopes by allowing one or two amino acid mismatches respectively (**Supplementary Figure 7**). Given the long sequence of Orf1ab, the majority of SARS-CoV-2 predicted epitopes shared with 229E, HKU1, NL63, OC43, and MERS-CoV were derived from Orf1ab, while those derived from other proteins were predominantly shared with SARS-CoV (**Supplementary Figure 7**).

While SARS-CoV and MERS-CoV shared the highest numbers of predicted epitopes with SARS-CoV-2, individuals have a greater exposure to common coronaviruses, 229E, HKU1, NL63, and OC43. To analyze the extent of cross-reactive responses that can be conferred by common coronaviruses, we investigated the number of unique SARS-CoV-2 predicted epitopes shared with either one of the four coronaviruses, namely 229E, HKU1, NL63, and OC43. There were 112 unique peptides out of 794 (14%) SARS-CoV-2 predicted epitopes shared with four common coronaviruses with a high sequence similarity (**Figure 4B**, **Supplementary Figure 8**). Among the 112 peptides, 21 (/794, 2.6%) had exact matches with SARS-CoV-2 peptides, while 36 (/794, 4.5%) and 55 (/794, 6.9%) peptides had one and two mismatches, respectively.

### Potential Risk of Self-Reactivity by SARS-CoV-2 Derived Predicted Epitopes

Viral peptides identical or highly similar to those of the host organism might not give rise to high-affinity T cell responses because most of the T cells binding with high affinity to self peptides are eliminated during thymic negative selection. However, some of these potentially self-reactive T cells escape thymic selection, and when primed in the context of an infection they might cause autoimmune response (19, 20). Thus, when looking for potential candidates for vaccine design, it is important to assess the sequence similarity of predicted epitopes with their best matching counterparts in human proteome.

**Figure 5:**
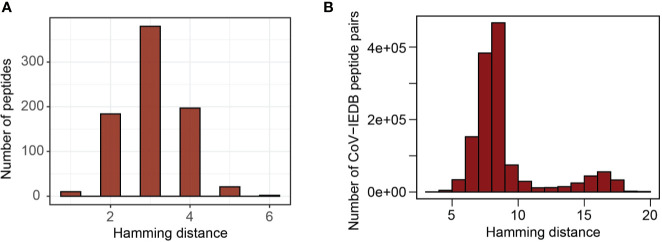
Sequence similarity with human proteome and influenza virus epitopes deposited in IEDB. **(A)** Distribution of hamming distance between SARS-CoV-2 derived peptides and human proteome counterparts (the region most similar to corresponding virus peptides). **(B)** Distribution of hamming distance between coronavirus derived peptides and all influenza virus epitopes deposited in IEDB.

By comparing predicted epitopes from SARS-CoV-2 with the human proteome, none of the predicted epitopes had identical match but we detected 10 and 184 epitopes with one and two amino acid mismatches respectively with their best matching human proteome counterparts (**Figure 5A**). The predicted epitopes differing by only one amino acid are listed in **Table 2**.

**Table 2:** Predicted epitopes from SARS-CoV-2 that are single amino acid variants of human proteome counterparts.

SARS-CoV-2 peptide	Human proteome pattern	SARS-CoV-2 protein	Human protein	Gene
FLALITLAT	LLALITLAT	ORF7a protein (ORF7a)	P28222|5HT1B	HTR1B
GDAALALLL	GAAALALLL	nucleocapsid phosphoprotein (N)	O14657|TOR1B, Q9BRX8|PXL2A	TOR1B, PRXL2A
GLPGTILRT	GQPGTILRT	orf1ab polyprotein (Orf1ab)	P51610|HCFC1	HCFC1
GLTVLPPLL	GLTVLPALL	surface glycoprotein (S)	P14672|GLUT4	SLC2A4
IPIGAGICA	IYIGAGICA	surface glycoprotein (S)	Q8TDQ0|HAVR2	HAVCR2
IVNSVLLFL	TVNSVLLFL	envelope protein (E)	O60518|RNBP6	RANBP6
QLSLPVLQV	QLLLPVLQV	orf1ab polyprotein (Orf1ab)	Q8IWE2|NXP20	FAM114A1
SLPINVIVF	SLPINVQVF	orf1ab polyprotein (Orf1ab)	Q12836|ZP4	ZP4
TPGSGVPVV	EPGSGVPVV	orf1ab polyprotein (Orf1ab)	P19021|AMD	PAM
VLPQLEQPY	VLPQNEQPY	orf1ab polyprotein (Orf1ab)	A2A3K4|PTPC1	PTPDC1

SARS-CoV-2 protein and human protein denote the proteins that SARS-CoV-2 peptide and human proteome patterns are derived, respectively. Note that only the best matching counterparts for each predicted SARS-CoV-2 epitopes are listed.

### Cross-Reactivity Between Coronavirus and Influenza Virus

In order to compare theoretical cross-reactivity between different coronaviruses with the one from unrelated viruses, we compared the 4,894 unique predicted epitopes from all coronavirus strains with 1334 unique MHC-I influenza virus-derived epitopes (1,298 influenza A virus and 36 influenza B virus) deposited in IEDB.

Due to a relative sequence dissimilarity between influenza virus and coronavirus, there were no peptides with identical match between two strains and all peptides were distinct by at least three amino acids (**Figure 5B**). This indicates a minimal potential for cross-protection against coronavirus, especially SARS-CoV-2, conferred by influenza virus.

Of note, among those with three amino acid differences, there were public predicted epitopes shared across multiple coronavirus strains as exemplified in **Table 3** (full list provided in **Supplementary Figure 9**). Despite a limited potential for cross-reactivity, these public peptides may cross-protect within coronavirus strains and given that they share a modest sequence similarity with epitopes derived from influenza virus, may pose a marginal potential to cross-react against influenza virus.

**Table 3:** Example of public predicted epitopes from coronavirus strains with modest sequence similarity with influenza virus epitopes.

Coronavirus	Influenza virus	Hammingdistance	Coronavirus strain	Influenza virus strain	Influenza virus protein
ALGGSVAIK	ILRGSVAHK	3	MERS	Influenza A virus	Nucleoprotein
ALGGSVAIK	ILRGSVAHK	3	OC43	Influenza A virus	Nucleoprotein
ALGGSVAIK	ILRGSVAHK	3	SARS-2	Influenza A virus	Nucleoprotein
ALALLLLDR	ALQLLLEV	3	SARS- 2	Influenza A virus	Nuclear export protein
ALALLLLDR	ALQLLLEV	3	SARS	Influenza A virus	Nuclear export protein
ALGGSVAIK	VLRGSVAHK	3	MERS	Influenza A virus (A/Netherlands/602/2009(H1N1))	Nucleoprotein
ALGGSVAIK	VLRGSVAHK	3	OC43	Influenza A virus (A/Netherlands/602/2009(H1N1))	Nucleoprotein
ALGGSVAIK	VLRGSVAHK	3	SARS -2	Influenza A virus (A/Netherlands/602/2009(H1N1))	Nucleoprotein

## Discussion

The severity and recurrence of coronavirus disease outbreaks pose ongoing global threat, and prompts the need for better understanding of potential cross-protection by prior infection of familial coronaviruses to mitigate the current spread and prevent future pandemics. Hereby, in continuation of our previous study to identify vaccine target for SARS-CoV-2 (21), we sought to determine the extent of antigen cross-reactivity amongst coronavirus strains.

Taking a step ahead of previous efforts to study potential immune recognition of SARS-CoV-2 by earlier infections of common coronaviruses based on MHC presentation predictions (22–25), we i) shortlisted predicted epitopes by taking immunogenicity potential of predicted binders into account, ii) validated the prediction by comparing with epitopes deposited in IEDB, iii) expanded the map of public predicted epitopes to accommodate up to two amino acid variants, and iv) analyzed sequence similarity against human proteome to eliminate self-peptides from vaccine targets. In comparison to Grifoni et al. (24, 26), the same algorithm has been adapted to predict HLA binding but moderate differences in HLA alleles and additional filtering strategies for immunogenicity led to differences in predicted epitope list. Detailed comparisons have been discussed in **Supplementary Figure 10**.

Due to the prevalence of disease caused by influenza virus and its tendency to gain mutations (antigenic drift) and reassortment among subtypes of virus (antigenic shift), there have been multiple attempts to develop generic vaccines protective against all influenza viruses to reduce the severity of infection and spread of disease (27, 28). Along with cross-reactivity among influenza virus strains, the cross-reactivity between coronavirus and influenza virus would benefit the community to combat recurrence of diseases caused by both strains. To aid design of vaccines targeting both coronaviruses and influenza viruses, we analyzed correlates of the predicted epitopes from coronaviruses with epitopes from influenza virus. However, due to a high sequence dissimilarity between these two virus families, there were no shared predicted epitopes, implying a limited potential of CD8 cross-protection against coronaviruses conferred by influenza virus. Nonetheless, we analyzed sequence similarity on 9-mers only and cannot neglect potential cross-reactivity conferred by shorter peptides or from CD4 + T cells.

Along with the *in silico* profile of predicted epitopes shared across coronaviruses, we evaluated the potential risk of self-reactivity imposed by high sequence similarity with the human proteome. Considering the reports of lung, heart, liver, intestine, genital and kidney failures by autoimmune disorders in COVID-19 patients (29), peptides from SARS-CoV-2 may carry high risk of immunopathology and should be carefully selected to proceed for vaccination.

In this study, we determined the potential immunogenicity by peptide sequence alone but other factors, such as turnover rate and expression level of parent proteins, may contribute to selection of immunodominant epitopes. For example, Bojkova et al. characterized changes in the protein level of SARS-CoV-2 infected Caco-2 cells, and showed 3–6 folds (in log2) increase in protein expressions after 20 h compared to 2 h post infection, with nucleocapsid and membrane proteins having the highest and the replicase polyprotein 1ab having the lowest fold change, respectively (30). Therefore, although fewer number of predicted epitopes were found from the membrane, nucleoprotein and spike proteins compared to the replicase polyprotein 1ab, their higher abundance might have a major impact in determining their immunodominance, and skew the immune response against their epitopes.

Comparing predicted epitopes with those characterized and deposited in IEDB, we could detect 64% of coronavirus IEDB epitopes from the prediction. In regards to the accuracy of predictive models utilized in this study, the algorithms to predict MHC presentation has matured significantly in the last decade by training with extensive datasets, especially for the most common HLA types. On the other hand, it is worth noting that predicting immunogenicity is challenging and not a fully solved problem. For example, these algorithms do not take into account other factors influencing the immunogenicity of a given epitope, such as abundance, expression pattern, and localization. Therefore, although the best performing models have been used for classifying immunogenicity, the predictions are suboptimal and should be taken with caution.

Along with the accuracy of predictive models, it is worth noting that classification thresholds of these algorithms have intrinsic tradeoff between sensitivity and specificity. In the case of SARS-CoV derived peptides, strong binding of HLA-A*02:01 has shown to correlate with stimulation of IFN*γ* secretion in the PBMC from HLA-A2 SARS-recovered donors *ex vivo* (31, 32), but we nevertheless cannot disregard potential immunogenicity from low-binding SARS-CoV-2 peptides. While we generated *in silico* map based on recommended thresholds of NetMHCpan and Repitope, stringent or relaxed thresholds can be applied based on the availability of resources for functional validations.

In this study, to validate our *in silico* identified epitopes, we compared our predicted epitopes with those characterized by *in vitro* T cell assays deposited in IEDB, and identified 22 out of 31 SARS-CoV IEDB epitopes matching our predictions. Please note that coronavirus-related peptides in IEDB are of varying lengths of up to 25aa, therefore multiple 9-mer epitopes could match with a single peptide in IEDB. In order to gauge the inevitable false negative rate, we compared peptides that didn’t pass our filters to IEDB peptides. We observed that 23 out of 43,732 predicted non-binders and 2 out of 11,202 predicted binder-non-immunogenic peptides matched with 10 coronavirus-derived epitopes in IEDB (9 from SARS-CoV and 1 from OC43). While the presence of multiple 9-mers matching with the same IEDB epitope is not surprising, exclusion of some matches by the MHC binding and immunogenicity filtering may facilitate determining the regions of peptide that are most likely to elicit the T cell response.

In a separate study, Nelde et al. (33) predicted 1,739 SARS-CoV-2 derived HLA-I peptides to be immunogenic based on integration of two algorithms, SYFPEITHI and NetMHCpan. They then selected 100 from 1,739 peptides to proceed for functional validation and reported 29/100 (29%) peptides as naturally occurring CD4+ or CD8+ T-cell epitopes. Of these 100 peptides, we compared only 9-mer peptides (59 peptides) with our predictions and found 39/59 (66%) peptides to agree with our predictions (**Supplementary Figure 11**). Out of these 39 common 9mers, we have identified 5 true positive and 34 true negative that leads to a predictive accuracy of 66%. However, one may notice that an additional filter also imposes a risk of increasing false negatives. Suitable filters should be decided based on the capacity of testing immunogenicity of peptides.

Notably, this study further demonstrated presence of T cell responses in unexposed individuals to 31% of their validated HLA class I epitopes, which hints to presence of CD8+ T cell cross-reactivity with common coronaviruses. In agreement with our prediction, Orf1ab was the protein most recognized by the class I epitopes they found in healthy donors, while membrane (M) and nucleocapsid (N) proteins were not recognized. This points to the potential cross-reactivity with other coronavirus strains and suggests the significance of conceptualizing the map of cross-reactive potential among coronavirus strains.

We believe that our comprehensive profile of private and public predicted epitopes across coronaviruses will assists biologists with targeted function validation, and facilitate design of vaccines capable of protecting against multiple prevalent virus strains. Further validations of the predicted profile would help estimate the extent of cross-protection and pave the way for better understanding of heterogeneity in the susceptibility and severity to the disease.

## Data Availability Statement

All datasets presented in this study are included in the article/**Supplementary Material**.

## Author Contributions

HK and GN conceived and designed the study. CL conceived and designed computational approaches, conducted data analysis, and generated figures and tables. PB conducted immunogenicity prediction. IW conducted MHC presentation prediction. CL MP, GO, AS, HK, and GN wrote the manuscript, and IW and PB contributed to methods. AS and HK supervised the project. All authors contributed to the article and approved the submitted version.

## Conflict of Interest

GO has served on advisory boards or holds consultancies or equity with Eli Lilly, Novartis, Janssen, Sanofi, Orbit Discovery, and UCB Pharma, and has undertaken clinical trials with Atopix, Regeneron/Sanofi, and Roche.

The remaining authors declare that the research was conducted in the absence of any commercial or financial relationships that could be construed as a potential conflict of interest.

## Supplementary Material

Supplementary Table 1 List of predicted scores for immunogenicity. Immunogenicity predicted by Repitope for all 9-mer peptides that have passed MHC presentation threshold.

Supplementary Table 2 List of predicted scores for MHC presentation and immunogenicity of 10-mer peptides.

Supplementary Table 3 List of predicted epitopes referenced in IEDB.

Supplementary Table 4 List of public predicted epitopes up to two mismatches. Expansion of shared epitopes to include up to two amino acid mismatches.
