# Supplementary material for: Potential CD8+ T Cell Cross-Reactivity Against SARS-CoV-2 Conferred by Other Coronavirus Strains
Source: Front Immunol. 2020 Nov 5;11:579480. doi: 10.3389/fimmu.2020.579480 (PMC7676914; doi:10.3389/fimmu.2020.579480)
Supplement: Supplementary file 1 [file DataSheet_1.pdf]

Supplementary Material for

Potential CD8+ T cell cross-reactivity against SARS-CoV-2 conferred by other coronavirus strains

Chloe H. Lee, Mariana Pereira Pinho, Paul R Buckley, Isaac B. Woodhouse, Graham Ogg, Alison Simmons, Giorgio Napolitani, Hashem Koohy

Supplementary Figure 1. Alignment of proteome sequences of spike protein among different coronavirus strains

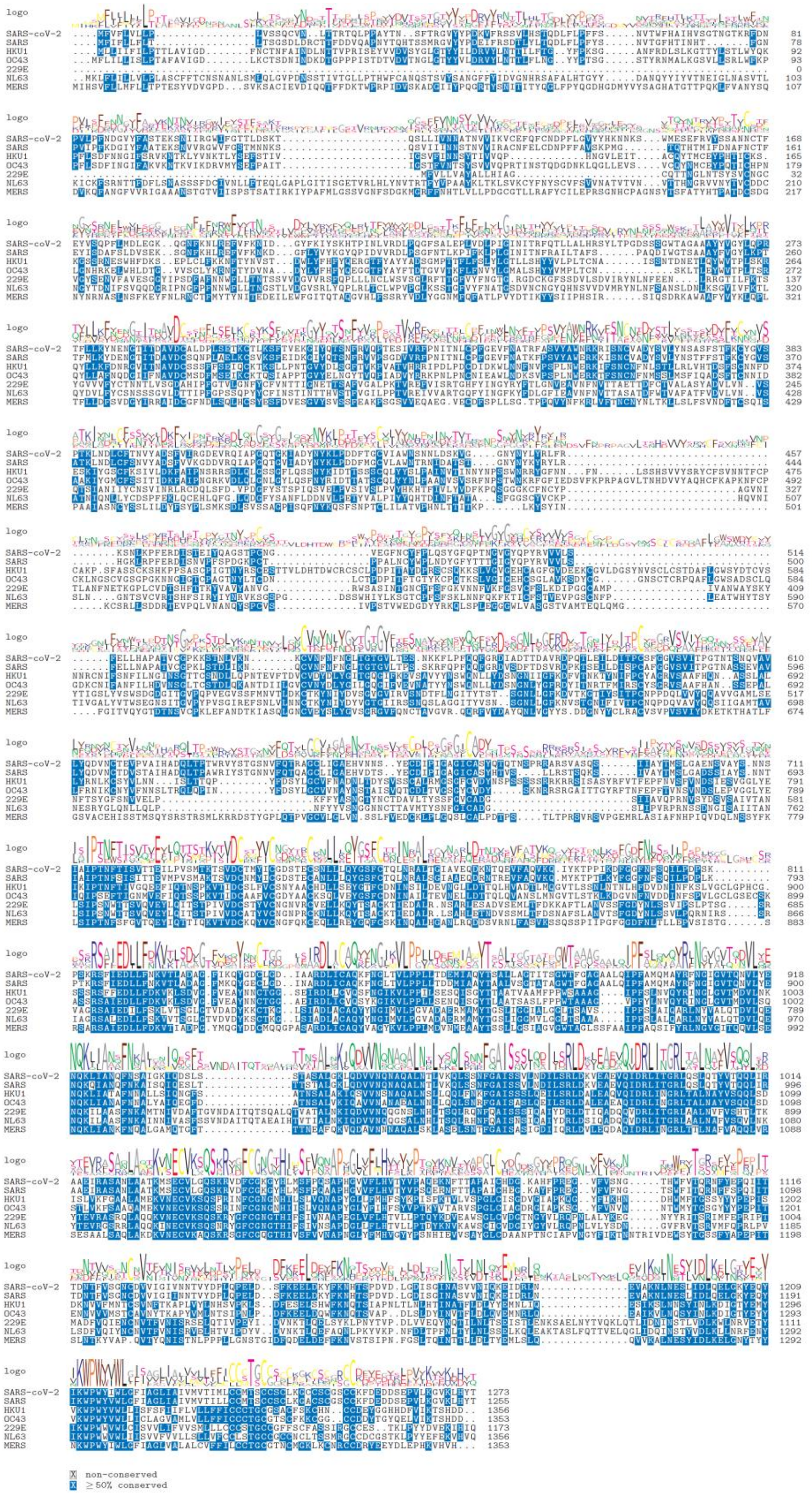

Supplementary Figure 2. Phylogenetic tree of encoded proteins, envelope protein (E), membrane protein (M), nucleocapsid protein (N) and replicase polyprotein (Orf1ab) for seven coronavirus strains. *The recurrent strain annotations are due to presence of two open reading frames annotated with the same functional protein.*

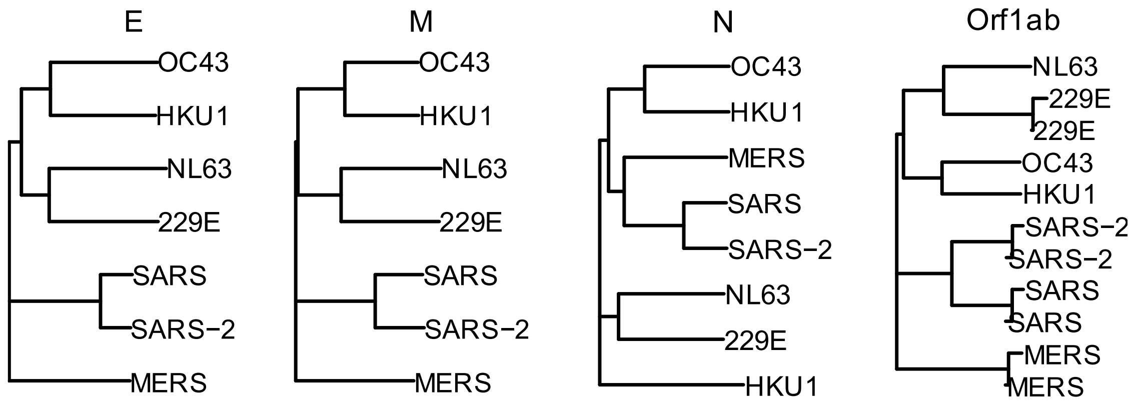

Supplementary Figure 3. A. Prevalence of 10 HLA alleles predicted to bind SARS-CoV-2 peptides. B. List of peptides predicted to bind 8 HLA alleles by NetMHCpan 4.0.

A. Prevalence of 10 HLA alleles in UK and USA population. The allele frequency describes range of frequencies from sub-populations of the denoted country, retrieved from Allele Frequency Net Database[1].

HLA allele	Country	Allele frequency
A*01:01	UK	0.2020 - 0.2080
	USA	0.0100 - 0.1646
A*02:01	UK	0.2740 - 0.3035
	USA	0.0349 - 0.2776
A*03:01	UK	0.1320 - 0.1430
	USA	0.0001 - 0.1399
A*24:02	UK	0.0610 - 0.0690
	USA	0.0336 - 0.3530
B*07:02	UK	0.1329 - 0.1730
	USA	0.0293 - 0.1306
B*40:01	UK	0.0330 - 0.0600
	USA	0.0001 - 0.1543
B*08:01	UK	0.0880 - 0.1620
	USA	0.0002 - 0.1144
C*07:02	UK	0.1484 - 0.1860
	USA	0.0664 - 0.2894
C*04:01	UK	0.0620 - 0.0857
	USA	0.0383 - 0.2279
C*07:01	UK	0.1618 - 0.1900
	USA	0.0047 - 0.1600

B. List of peptides predicted to bind 8 HLA alleles by NetMHCpan 4.0.

peptide	strain	ProteinID
FVNDKITEF	229E	Orf1ab
YFDGVKTVL	229E	Orf1ab
YVYKRGMQL	HKU1	E
YVDPKYQVI	MERS	Orf1ab
MLSDNVVAF	NL63	Orf1ab
HLYNVTRTF	NL63	S
KQMYKTPTL	SARS	S

Supplementary Figure 4. (Top) Number of peptides predicted to be immunogenic by Repitope, facet by HLA alleles predicted to bind via NetMHCpan. (Bottom) Number of predicted epitopes predicted to be N number of HLA alleles for each coronavirus strain.

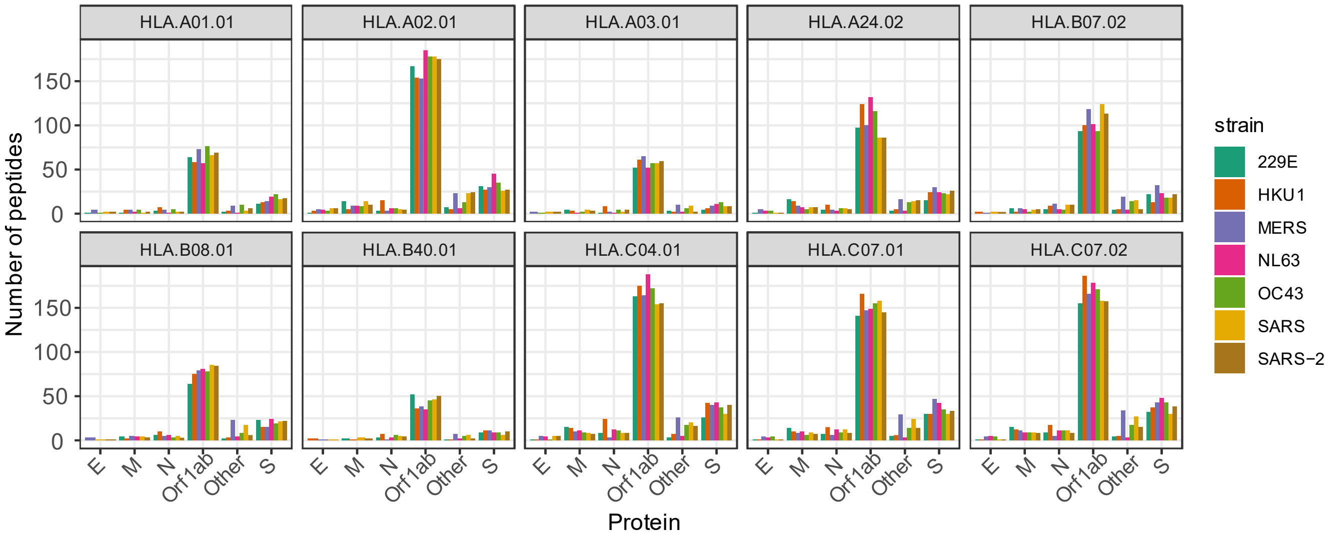

N of HLA	229E	HKU1	MERS	NL63	OC43	SARS	SARS- 2
**1**	391	406	459	414	428	460	438
**2**	128	116	160	136	137	169	152
**3**	65	91	75	93	79	71	71
**4**	71	91	80	86	86	67	68
**5**	44	37	39	35	43	52	48
**6**	13	20	26	16	20	9	14
**7**	1	2	5	4	3	5	3
**8**	0	0	0	1	0	0	0

Supplementary Figure 5. Public and private predicted epitopes after MHC binding and immunogenicity predictions. (A) UpSet diagram illustrating private and public 9-mer peptides from all encoded proteins after NetMHCpan and Repitope predictions. (B) Venn diagram illustrating private and public 9-mer peptides of SARS-CoV-2 from all encoded proteins. (C) Venn diagram illustrating private and public 9-mer peptides from a complete set of peptides to after MHC presentation prediction by NetMHCpan and immunogenicity prediction by Repitope. The encoded proteins are replicase polyprotein (Orf1ab), nucleocapsid protein (N), envelop protein (E), membrane protein (M) and remaining encoded proteins combined (Other).

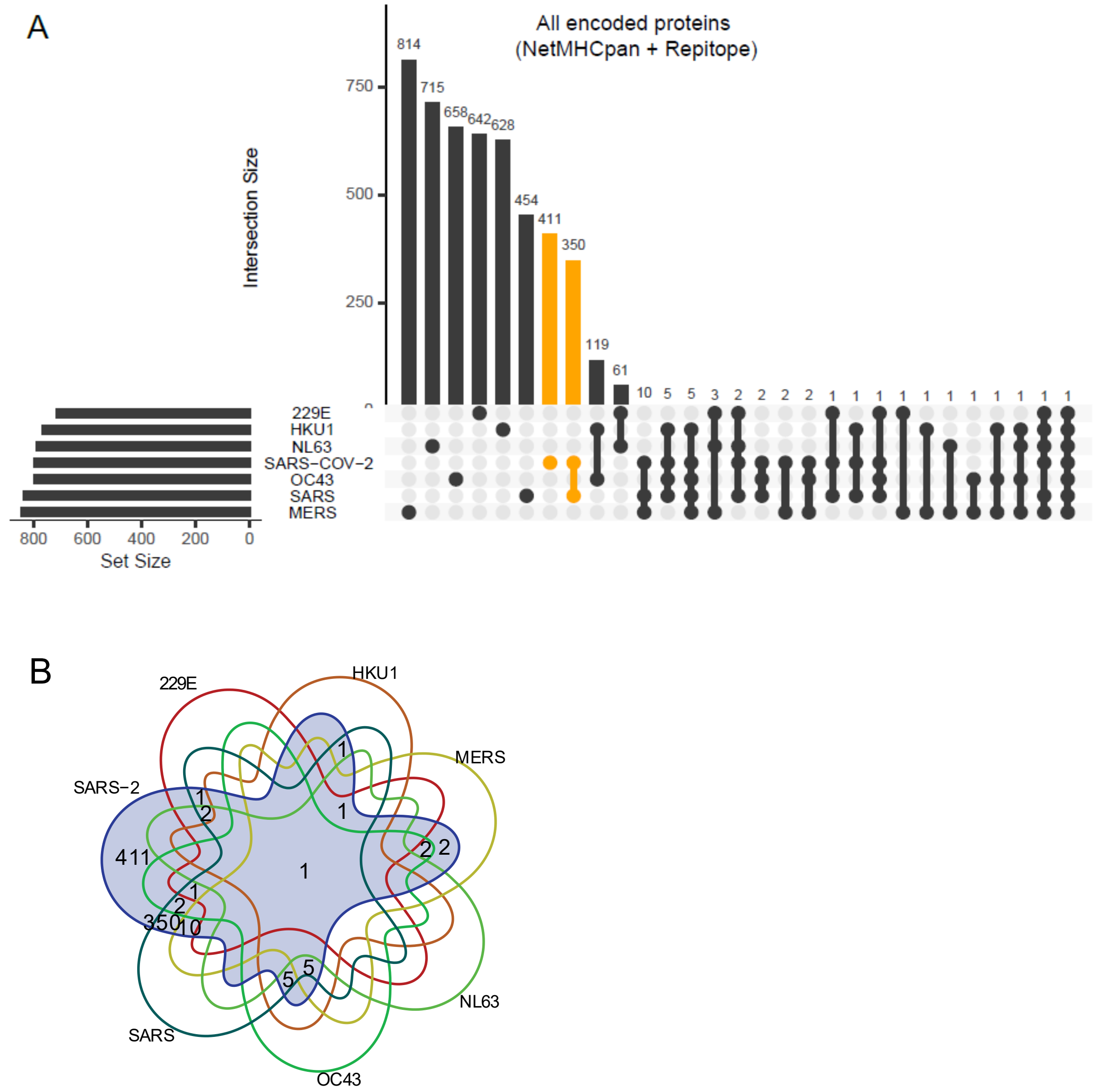

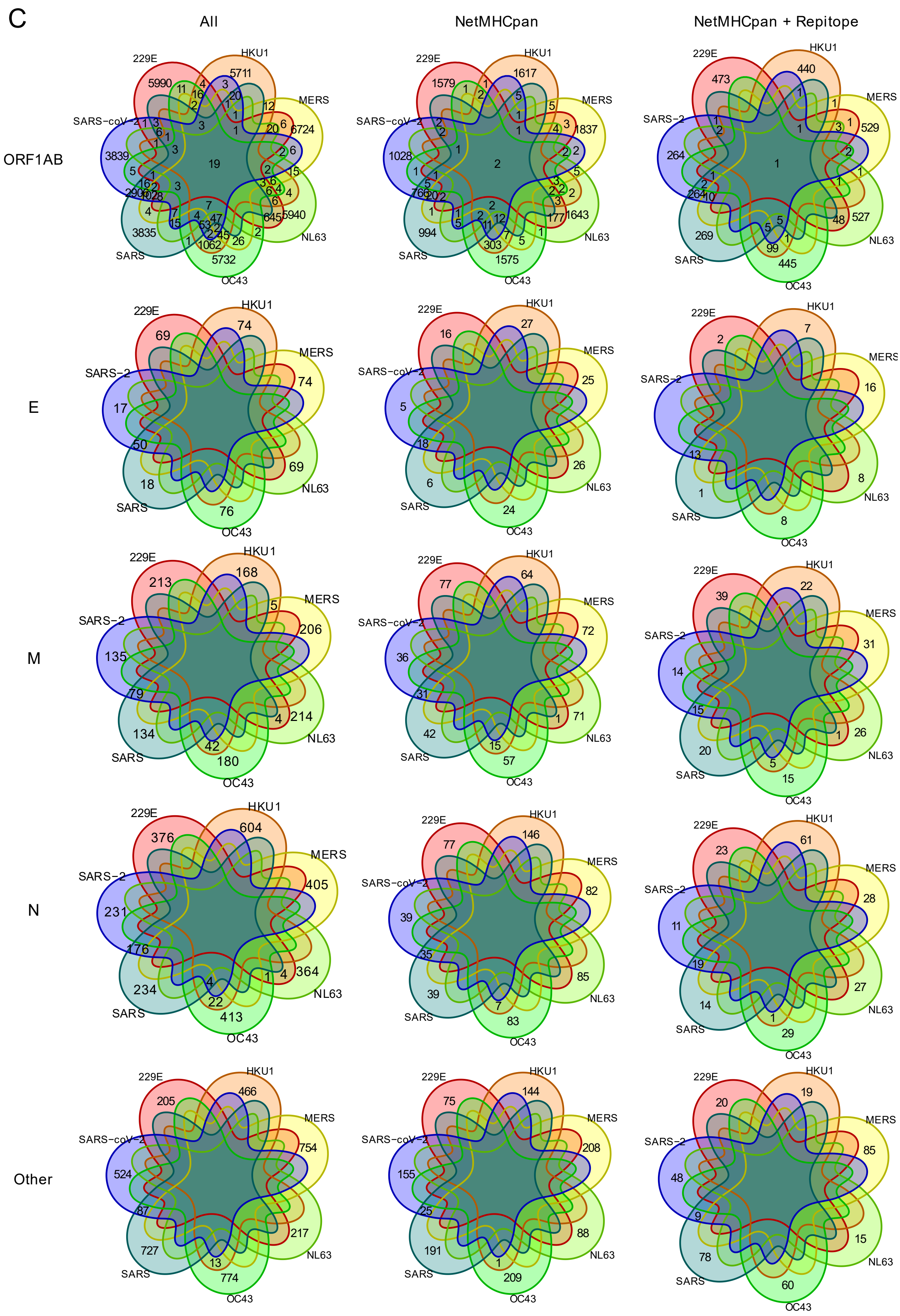
C
All
NetMHCpan
NetMHCpan + Repitope
ORF1AB
E
M
N
Other
229E
HKU1
MERS
NL63
OC43
SARS
SARS−coV−2
SARS−2

Supplementary Figure 6. Prediction of MHC binding and immunogenicity for 10-mer peptides. A. Venn diagram illustrating private and public 10-mer peptides from a complete set of peptides to after MHC presentation prediction by NetMHCpan and immunogenicity prediction by Repitope. The encoded proteins are replicase polyprotein (Orf1ab), nucleocapsid protein (N), envelop protein (E), membrane protein (M) and remaining encoded proteins combined (Other). B. Number of 10-mer peptides from each coronavirus strains predicted to bind corresponding HLA alleles and be immunogenic.

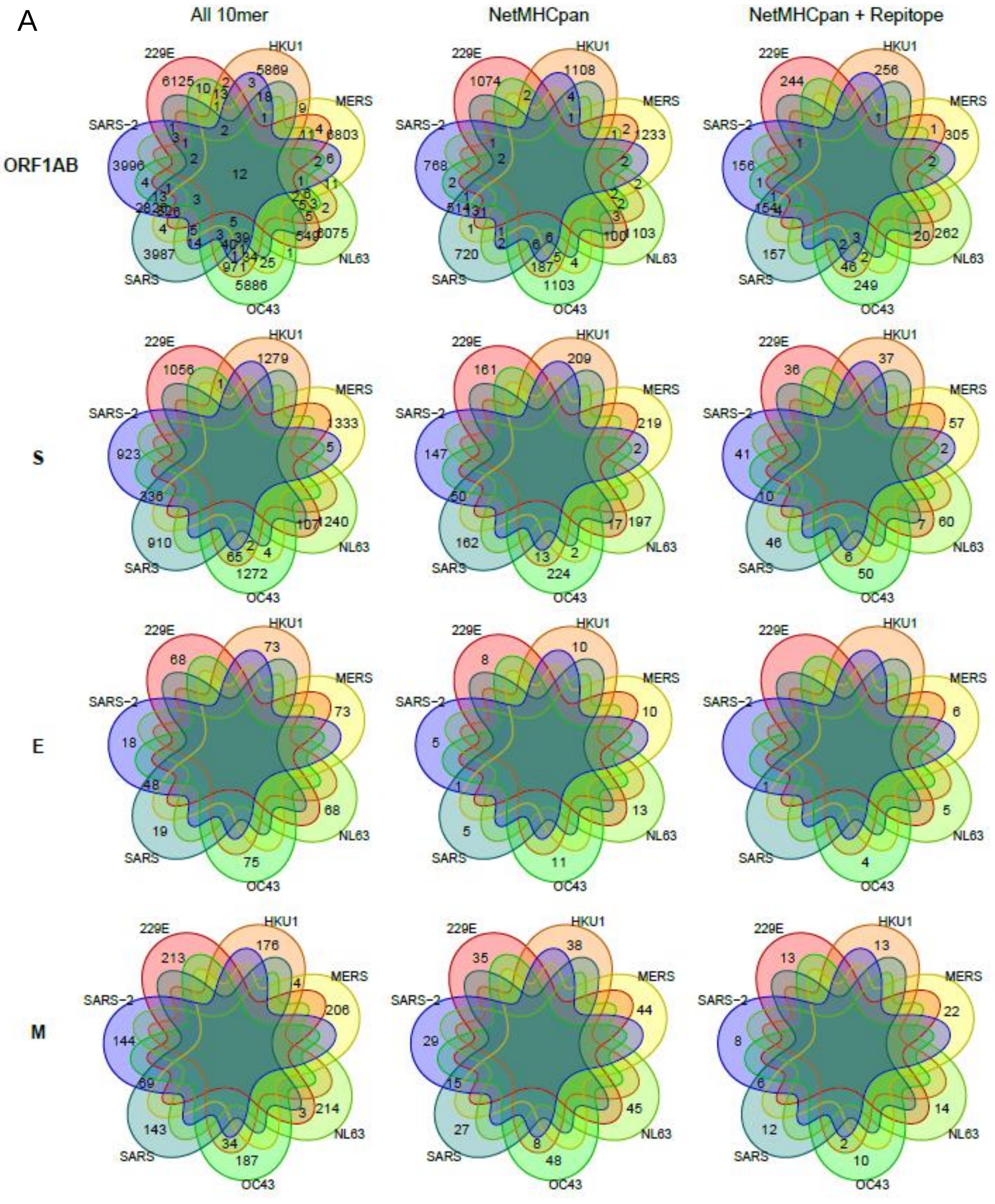

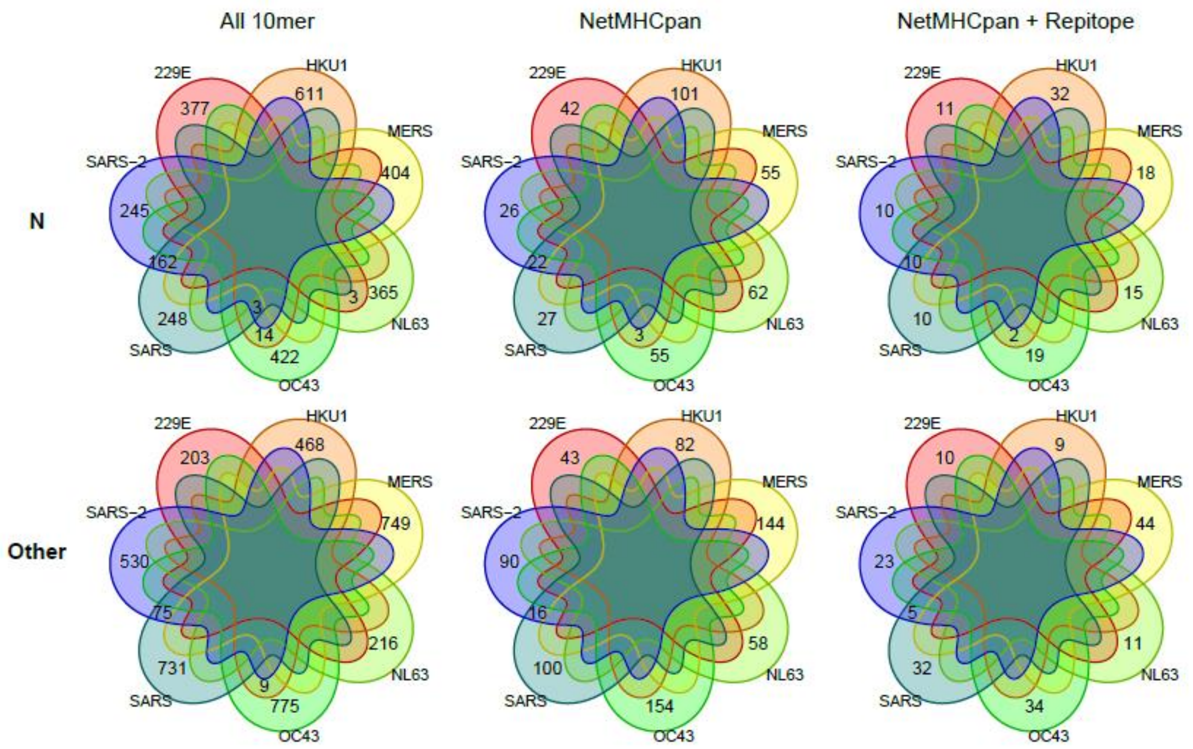

All 10mer
NetMHCpan
NetMHCpan + Repitope
N
Other
229E
HKU1
MERS
SARS−2
NL63
SARS
OC43
377
611
404
245
162
3
365
248
3
14
422
42
101
55
26
22
62
27
3
55
11
32
18
10
10
15
10
2
19
203
468
749
530
75
216
731
9
775
43
82
144
90
16
58
100
154
10
9
44
23
5
11
32
34

B

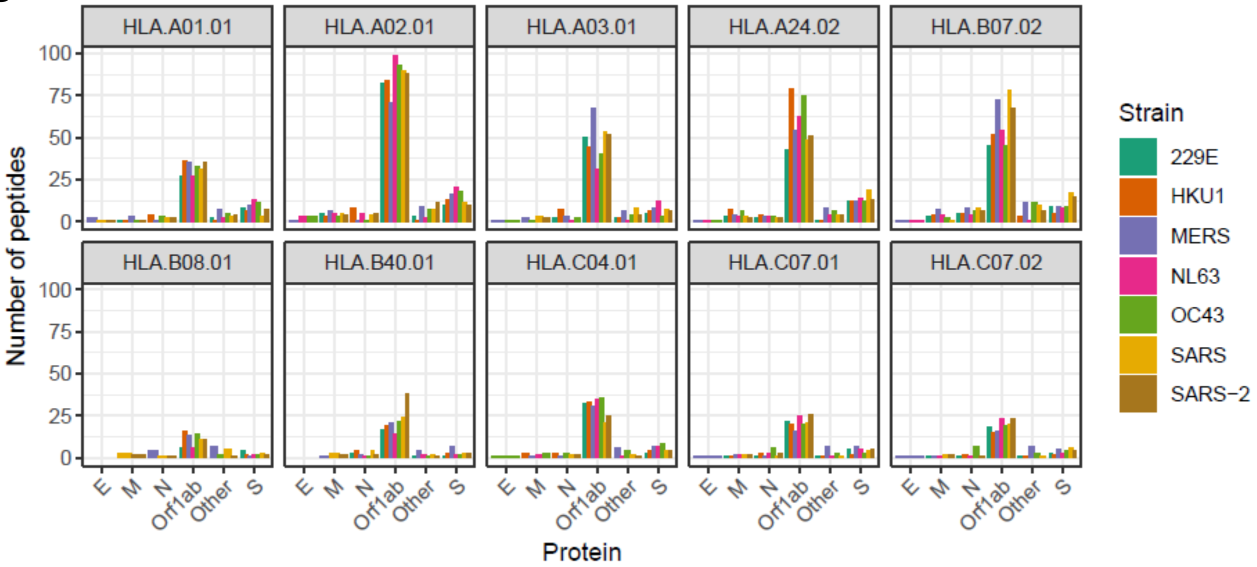

HLA.A01.01
HLA.A02.01
HLA.A03.01
HLA.A24.02
HLA.B07.02
HLA.B08.01
HLA.B40.01
HLA.C04.01
HLA.C07.01
HLA.C07.02
Number of peptides
100
75
50
25
0
E
M
N
Orf1ab
Other
S
Protein
Strain
229E
HKU1
MERS
NL63
OC43
SARS
SARS−2

Supplementary Figure 7. Number of shared predicted epitopes between SARS-CoV-2 and other coronavirus strains by allowing up to two mismatches. (Top) Number of shared predicted epitopes faceted by their protein IDs out of 559 replicase polyprotein (Orf1ab), 106 spike protein (S), 13 envelop protein (E), 29 membrane protein (M), 30 nucleocapsid protein (N) and 57 remaining proteins (Other) SARS-CoV-2 predicted epitopes. (Bottom) Table illustrating number of SARS-CoV-2 predicted epitopes shared with other coronaviruses. This table includes duplicated peptides that may be shared across multiple coronavirus strains, i.e. peptides shared across SARS-CoV-2, SARS-CoV and MERS-CoV are counted in both SARS and MERS. Numbers in brackets reflect percentage of shared peptides out of total 794 SARS-CoV-2 predicted epitopes.

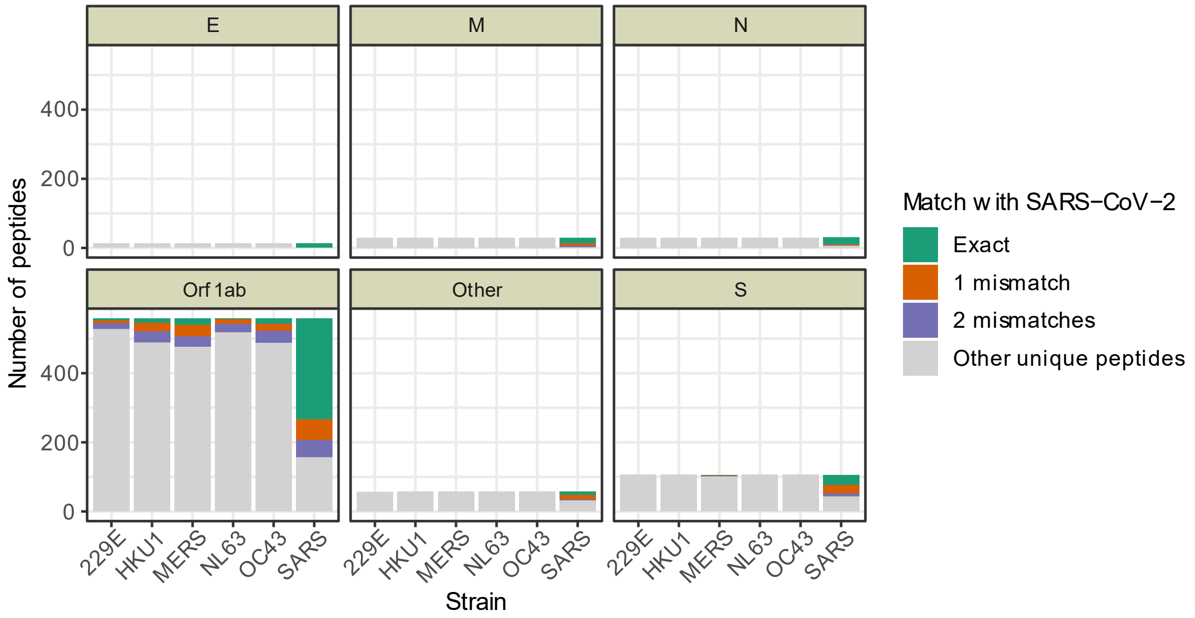

Strain	Match with 794 SARS-CoV-2 predicted epitopes (%)		
	**Exact**	**1 mismatch**	**2 mismatches**
229E	6 (0.76)	8 (1.01)	21 (2.64)
HKU1	13 (1.64)	27 (3.40)	34 (4.28)
MERS	21 (2.64)	37 (4.66)	33 (4.16)
NL63	4 (0.50)	12 (1.51)	27 (3.40)
OC43	16 (2.02)	24 (3.02)	36 (4.53)
SARS	379 (47.73)	108 (13.60)	68 (8.56)

Supplementary Figure 8. Number of SARS-CoV-2 predicted epitopes shared with any common coronaviruses, 229E, HKU1, NL63, OC43, by allowing up to two mismatches.

Match with common coronaviruses	Number of SARS-CoV-2 predicted epitopes
Exact	21
1 mismatch	36
2 mismatches	55
Other unique peptides	682
Total	794

Supplementary Figure 9. List of predicted epitopes from coronavirus and epitopes from influenza virus deposited in IEDB with a modest sequence similarity.

**Coronavirus peptides**	**Influenza virus epitopes**	**coronavirus**	**IV Parent.Protein from IEDB**	**Organism.Name from IEDB**
AEIRDLICV	AEIEDLIFL	OC43	Nucleoprotein	Influenza A virus H3N2 (A/Netherlands/9/03 (H3N2))
AEIRDLICV	AEIEDLIFS	OC43	Nucleoprotein	Influenza A virus (A/Bilthoven/4791/1981(H3N2))
SLGGSVAIK	ILRGSVAHK	HKU1	Nucleoprotein	Influenza A virus
ALGGSVAIK	ILRGSVAHK	MERS	Nucleoprotein	Influenza A virus
ALGGSVAIK	ILRGSVAHK	OC43	Nucleoprotein	Influenza A virus
ALGGSVAIK	ILRGSVAHK	SARS-2	Nucleoprotein	Influenza A virus
NELSRVLGL	NMLSTVLGV	SARS-2	RNA-directed RNA polymerase catalytic subunit	Influenza A virus (A/Puerto Rico/8/1934(H1N1))
MGLFRRRSV	RGLQRRRFVQNALNGNG	OC43	Matrix protein 1	Influenza A virus (A/Puerto Rico/8/1934(H1N1))
AEIRDLICV	AEIEDLIFLA	OC43	Nucleoprotein	Influenza A virus
ALALLLLDR	ALQLLLEV	SARS-2	Nuclear export protein	Influenza A virus
ALALLLLDR	ALQLLLEV	SARS	Nuclear export protein	Influenza A virus
FLDLRTSCF	FEDLRVSSF	MERS	Nucleoprotein	Influenza A virus
NELSRVLGL	FNMLSTVLGV	SARS-2	RNA-directed RNA polymerase catalytic subunit	Influenza A virus
SLLNLKLRA	LLFLKVPA	MERS	RNA-directed RNA polymerase catalytic subunit	Influenza A virus
LSLLKVTAF	LLFLKVPA	SARS	RNA-directed RNA polymerase catalytic subunit	Influenza A virus
SPLFLIVAA	LLFLKVPA	SARS	RNA-directed RNA polymerase catalytic subunit	Influenza A virus
FLDLRTSCF	VWMACHSAAFEDLRVSSF	MERS	Nucleoprotein	Influenza A virus (A/Vietnam/CL26/2004(H5N1))
EQAAAAMYK	SEQAAEAMEV	229E	Matrix protein 1	Influenza A virus
SLGGSVAIK	VLRGSVAHK	HKU1	Nucleoprotein	Influenza A virus (A/Netherlands/602/2009(H1N1))
ALGGSVAIK	VLRGSVAHK	MERS	Nucleoprotein	Influenza A virus (A/Netherlands/602/2009(H1N1))
ALGGSVAIK	VLRGSVAHK	OC43	Nucleoprotein	Influenza A virus (A/Netherlands/602/2009(H1N1))
ALGGSVAIK	VLRGSVAHK	SARS-2	Nucleoprotein	Influenza A virus (A/Netherlands/602/2009(H1N1))
IIGGLHLLI	IIGILHLIL	OC43	Matrix protein 2	Influenza A virus

Supplementary Figure 10. Compare predicted epitope list from Grifoni et al.[2] with predicted epitope list. A. Comparison between Grifoni et al. predicted epitope list with peptides predicted to bind at least one HLA alleles (Supplementary Data 1). B. Comparison between Grifoni et al. predicted epitope list with peptides predicted to be immunogenic (Supplementary Data 2).

Grifoni *et al.* predicted epitopes using NetMHCpan 4.0 EL algorithm alone on 12 most prominent HLA alleles (HLA-A*01:01, HLA-A*02:01, HLA-A*03:01, HLA-A*11:01, HLA-A*23:01, HLA-A*24:02, HLA-B*07:02, HLA-B*08:01, HLA-B*35:01, HLA-B*40:01, HLA-B*44:02, HLA-B*44:03), and selected 1% scoring peptides. We've used the same NetMHCpan 4.0 EL algorithm, but set rank score <= 2.0 (recommended by the developer) as the threshold. However, the difference in predicted binder list (Figure A) would mostly be due to different list of HLA alleles subjected for analysis, where our HLA-A and -B alleles overlapped with Grifoni et al 's 12 HLA alleles but we added HLA-C alleles as a part of our analysis (HLA-A*01:01, 02:01, 03:01, 24:02, HLA-B*07:02, 40:01, 08:01 and HLA-C*07:02, 04:01, 07:01). In addition to NetMHCpan 4.0 to predict MHC binding, we applied Repitope to predict immunogenicity (Figure B). As discussed in comparative study with Nelde *et al.*[3], in Discussion, we have seen that while additional filtering step may result in reduced sensitivity by the expense of greater specificity, the overall predictive accuracy improved.

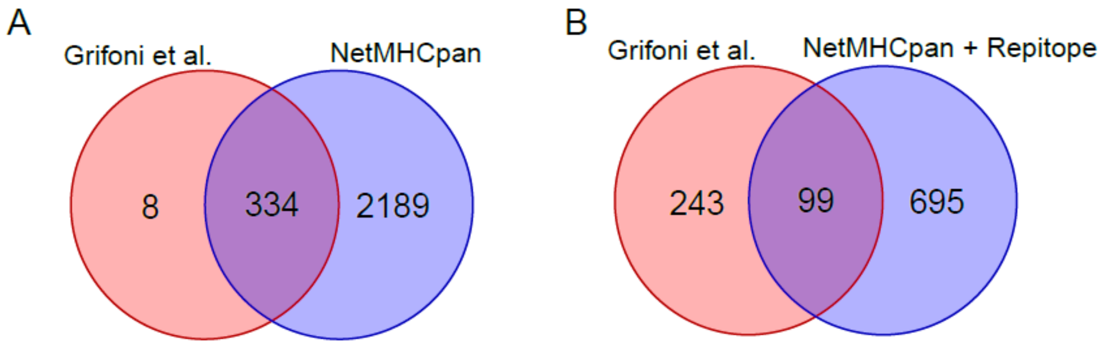

Supplementary Figure 11. Compare functional validations from Nelde et al.[3] with predicted epitope list. A. Confusion matrix illustrating predictive accuracy of our prediction by comparing $CD4^+$ or $CD8^+$ T cell immunogenicity characterized by IFNγ ELISPOT screening. B. List of 9-mer peptides from Nelde et al. compared with predictions. Immunogenicity refers to immunogenicity by either CD4+ or CD8+ T cells characterized by IFNγ ELISPOT screening. Predicted immunogenicity refers to qualitative classification of immunogenicity based on predicted score of Repitope. Predicted HLA alleles lists all HLA alleles out of 10 HLA alleles (HLA-A*0101, 0201, 0301, 2402, HLA-B*0702, 4001, 0801 and HLA-C*0702, 0401, 0701 alleles) predicted to bind the particular peptide by NetMHCpan 4.0 prediction.

A. Confusion Matrix and Statistics

	**Functional validation**	
**Predictions**	Negative	Positive
Negative	34	9
Positive	11	5

Accuracy : 0.661
95% CI : (0.5261, 0.7792)
Sensitivity : 0.7556
Specificity : 0.3571
Prevalence : 0.7627
Detection Rate : 0.5763
Detection Prevalence : 0.7288
Balanced Accuracy : 0.5563

B. List of 9-mer peptides from Nelde et al. compared with predictions.

Peptide	Immunogenicity	Predicted Immunogenicity	Immunogenicity Predicted Score	Predicted HLA allele
KLFAAETLK	Positive	Positive	0.38296	HLA.A03.01
VYIGDPAQL	Positive	Positive	0.4416	HLA.A24.02|HLA.C04.01|HLA.C07.01|HLA.C07.02
QYIKWPWYI	Positive	Positive	0.43888	HLA.A24.02|HLA.C04.01|HLA.C07.01|HLA.C07.02
FPRGQGVPI	Positive	Positive	0.48152	HLA.B07.02|HLA.B08.01
QRNAPRITF	Positive	Positive	0.4076	HLA.B07.02|HLA.C04.01|HLA.C07.01|HLA.C07.02
AGDSGFAAY	Negative	Positive	0.39248	HLA.A01.01|HLA.C04.01|HLA.C07.01|HLA.C07.02
FLLPSLATV	Negative	Positive	0.52576	HLA.A02.01|HLA.C04.01|HLA.C07.01|HLA.C07.02
FIAGLIAIV	Negative	Positive	0.90184	HLA.A02.01
FLAFVVFLL	Negative	Positive	0.39192	HLA.A02.01
KLLEQWNLV	Negative	Positive	0.38912	HLA.A02.01
NIVNVSLVK	Negative	Positive	0.38504	HLA.A03.01
ASMPTTIAK	Negative	Positive	0.39936	HLA.A03.01
SVLNDILSR	Negative	Positive	0.44368	HLA.A03.01
ASAFFGMSR	Negative	Positive	0.38808	HLA.A03.01
TPINLVRDL	Negative	Positive	0.44528	HLA.B07.02|HLA.B08.01
VRFPNITNL	Negative	Positive	0.39592	HLA.B08.01|HLA.B40.01|HLA.C04.01|HLA.C07.01|HLA.C07.02
LTDEMIAQY	Positive	Negative	0.22984	HLA.A01.01|HLA.C04.01|HLA.C07.01|HLA.C07.02
ALSKGVHFV	Positive	Negative	0.3552	HLA.A02.01|HLA.C04.01
VYFLQSINF	Positive	Negative	0.22664	HLA.A24.02|HLA.C04.01|HLA.C07.01|HLA.C07.02
TPKYKFVRI	Positive	Negative	0.23216	HLA.B07.02|HLA.B08.01
FVKHKHAFL	Positive	Negative	0.2844	HLA.B08.01|HLA.C07.01|HLA.C07.02
DLKGKYVQI	Positive	Negative	0.20912	HLA.B08.01
EAFEKMVSL	Positive	Negative	0.24656	HLA.B07.02|HLA.B08.01|HLA.C04.01|HLA.C07.01|HLA.C07.02
YYQLYSTQL	Positive	Negative	0.34944	HLA.A24.02|HLA.B08.01|HLA.C04.01|HLA.C07.01|HLA.C07.02
NRFLYIIKL	Positive	Negative	0.1784	HLA.B08.01|HLA.B40.01|HLA.C04.01|HLA.C07.01|HLA.C07.02
RQEEVQELY	Negative	Negative	0.1208	HLA.A01.01|HLA.C04.01
SPDDQIGYY	Negative	Negative	0.20296	HLA.A01.01|HLA.B07.02|HLA.C04.01|HLA.C07.01|HLA.C07.02
YIDIGNYTV	Negative	Negative	0.23104	HLA.A01.01|HLA.A02.01|HLA.C04.01|HLA.C07.01|HLA.C07.02
RLFRKSNLK	Negative	Negative	0.27352	HLA.A03.01
RIAGHHLGR	Negative	Negative	0.36184	HLA.A03.01
NLIIKNLSK	Negative	Negative	0.30688	HLA.A03.01
VTYVPAQEK	Negative	Negative	0.17376	HLA.A03.01
ASKIITLKK	Negative	Negative	0.25528	HLA.A03.01
NLIIKNLSK	Negative	Negative	0.30688	HLA.A03.01
GVKHVYQLR	Negative	Negative	0.21416	HLA.A03.01
SSTASALGK	Negative	Negative	0.2852	HLA.A03.01
APFLYLYAL	Negative	Negative	0.34688	HLA.B07.02|HLA.B08.01

EPKLGSLVV	Negative	Negative	0.30968	HLA.B07.02|HLA.B08.01
YLKLRSDVL	Negative	Negative	0.21976	HLA.B08.01|HLA.C04.01|HLA.C07.01|HLA.C07.02
EPVLKGVKL	Negative	Negative	0.22752	HLA.B07.02|HLA.B08.01
IIKNLSKSL	Negative	Negative	0.22776	HLA.B07.02|HLA.B08.01
TLDSKTQSL	Negative	Negative	0.16552	HLA.A01.01|HLA.A02.01|HLA.B07.02|HLA.B08.01|HLA.C04.01|HLA.C07.01|HLA.C07.02
VPMEKLKTL	Negative	Negative	0.31872	HLA.B07.02|HLA.B08.01|HLA.C04.01|HLA.C07.01|HLA.C07.02
GAKLKALNL	Negative	Negative	0.34312	HLA.B08.01
YQKVGMQKY	Negative	Negative	0.24856	HLA.A01.01|HLA.C07.01|HLA.C07.02
VLKGVKLHY	Negative	Negative	0.24	HLA.A01.01|HLA.A03.01
FLYLYALVY	Negative	Negative	0.3372	HLA.A01.01|HLA.A03.01
LVKPSFYVY	Negative	Negative	0.19944	HLA.A01.01|HLA.A03.01|HLA.C07.01|HLA.C07.02
WLSYFIASF	Negative	Negative		Predicted nonbinder
KVSIWNLDY	Negative	Negative	0.18952	HLA.A01.01|HLA.A03.01
RQEEVQELY	Negative	Negative	0.1208	HLA.A01.01|HLA.C04.01
IQYIDIGNY	Negative	Negative	0.2272	HLA.A03.01
LLNKHIDAY	Negative	Negative	0.21096	HLA.A01.01|HLA.A03.01
NYMPYFFTL	Negative	Negative	0.2984	HLA.A24.02|HLA.B08.01|HLA.C04.01|HLA.C07.01|HLA.C07.02
IRQEEVQEL	Negative	Negative	0.2604	HLA.C04.01|HLA.C07.01|HLA.C07.02
EYHDVRVVL	Negative	Negative	0.20432	HLA.A24.02|HLA.B08.01|HLA.C04.01|HLA.C07.01|HLA.C07.02
KKADETQAL	Negative	Negative	0.26312	HLA.C07.01|HLA.C07.02
VYDPLQPEL	Negative	Negative	0.32408	HLA.A24.02|HLA.C04.01|HLA.C07.01|HLA.C07.02
IYNDKVAGF	Negative	Negative	0.19256	HLA.A24.02|HLA.C04.01|HLA.C07.01|HLA.C07.02

References

1. Gonzalez-Galarza, F. F., McCabe, A., Santos, E. J. M. dos, Jones, J., Takeshita, L., Ortega-Rivera, N. D., Cid-Pavon, G. M. D., Ramsbottom, K., Ghattaoraya, G., Alfirevic, A., Middleton, D. & Jones, A. R. Allele frequency net database (AFND) 2020 update: gold-standard data classification, open access genotype data and new query tools. *Nucleic Acids Res* **48**, D783–D788 (2020).

2. Grifoni, A., Sidney, J., Zhang, Y., Scheuermann, R. H., Peters, B. & Sette, A. A Sequence Homology and Bioinformatic Approach Can Predict Candidate Targets for Immune Responses to SARS-CoV-2. *Cell Host & Microbe* **27**, 671-680.e2 (2020).

3. Nelde, A., Bilich, T., Heitmann, J. S., Maringer, Y., Salih, H. R., Roerden, M., Lübke, M., Bauer, J., Rieth, J., Wacker, M., Peter, A., Hörber, S., Traenkle, B., Kaiser, P. D., Rothbauer, U., Becker, M., Junker, D., Krause, G., Strengert, M., Schneiderhan-Marra, N., Templin, M. F., Joos, T. O., Kowalewski, D. J., Stos-Zweifel, V., Fehr, M., Rabsteyn, A., Mirakaj, V., Karbach, J., Jäger, E., Graf, M., Gruber, L.-C., Rachfalski, D., Preuß, B., Hagelstein, I., Märklin, M., Bakchoul, T., Gouttefangeas, C., Kohlbacher, O., Klein, R., Stevanović, S., Rammensee, H.-G. & Walz, J. S. SARS-CoV-2-derived peptides define heterologous and COVID-19-induced T cell recognition. *Nature Immunology* 1–12 (2020) doi:10.1038/s41590-020-00808-x.
